# EasyClone 2.0: expanded toolkit of integrative vectors for stable gene expression in industrial *Saccharomyces cerevisiae* strains

**DOI:** 10.1007/s10295-015-1684-8

**Published:** 2015-09-16

**Authors:** Vratislav Stovicek, Gheorghe M. Borja, Jochen Forster, Irina Borodina

**Affiliations:** The Novo Nordisk Foundation Center for Biosustainability, Technical University of Denmark, Kogle Allé 6, 2970 Hørsholm, Denmark

**Keywords:** Industrial yeast, Integrative vectors, Heterologous gene expression, Metabolic engineering, Xylose utilization

## Abstract

*Saccharomyces cerevisiae* is one of the key cell factories for production of chemicals and active pharmaceuticals. For large-scale fermentations, particularly in biorefinery applications, it is desirable to use stress-tolerant industrial strains. However, such strains are less amenable for metabolic engineering than the standard laboratory strains. To enable easy delivery and overexpression of genes in a wide range of industrial *S. cerevisiae* strains, we constructed a set of integrative vectors with long homology arms and dominant selection markers. The vectors integrate into previously validated chromosomal locations via double cross-over and result in homogenous stable expression of the integrated genes, as shown for several unrelated industrial strains. Cre-mediated marker rescue is possible for removing markers positioned on different chromosomes. To demonstrate the applicability of the presented vector set for metabolic engineering of industrial yeast, we constructed xylose-utilizing strains overexpressing xylose isomerase, xylose transporter and five genes of the pentose phosphate pathway.

**Electronic supplementary material:**

The online version of this article (doi:10.1007/s10295-015-1684-8) contains supplementary material, which is available to authorized users.

## Introduction

The yeast *Saccharomyces cerevisiae* is an important industrial host for production of bio-based chemicals [27]. The products manufactured by fermentation of natural or genetically engineered *S. cerevisiae* span from fuels (ethanol, isobutanol) and bulk chemicals (succinic acid) to enzymes (invertase) [4, 29] and nutraceutical (resveratrol) [20] and pharmaceutical ingredients (insulin) [28]. For production of high-volume low-cost fuels and chemicals, it is essential that the fermentation process shows high titer, rate and yield of a product on the substrate and thus extensive development of the cell factory is necessary to reach these parameters. Metabolic engineering undertakes a rational approach to redirect the metabolic fluxes towards the desired product by targeted manipulation of the cell’s genome involving insertion of heterologous pathways, overexpression and downregulation of multiple genes, construction of synthetic regulatory circuits, etc. [14, 24]. Multiple rounds of genetic engineering are commonly required until the cell with desired properties is obtained. For large-scale industrial applications, robust industrial strains are preferred as the hosts. In biorefineries, yeast cells must perform under various stresses, such as fluctuating temperature and pH, high osmotic pressure and presence of inhibitors coming from biomass hydrolysis [1, 35]. Industrial strains are, however, more difficult to genetically manipulate than the laboratory strains. Such strains are typically prototrophic, diploid, polyploid or even aneuploid and often exhibit low transformation efficiencies and lower levels of homologous recombination [44]. The genetic engineering toolbox for manipulation of industrial strains is currently very limited when compared to the tools for well-studied laboratory strains [2].

A widely used way for introduction of heterologous DNA sequence is by the use of autonomously replicating vectors, low copy centromeric and high copy episomal, or integrative vectors [45]. The use of autonomously replicating vectors requires selective pressure and results, especially in case of episomal vectors, in segregational instability and population variation. Thus, the expression of the genes often fluctuates in the cell population [3]. Moreover, when it comes to expression of multiple genes, the maintenance of such vectors in amount of two or more at the same time in single cell is not feasible [11]. If multiple genes are cloned into a single episomal vector, gene loss may occur by homologous recombination [48]. Therefore, chromosomal integration of genetic material is the preferred method for generating stable strains. The ease of homologous recombination in yeast led to the development of cloning-free methods using in vivo assembly of PCR-generated multiple DNA fragments and their insertion into the genome [41]. This can even be facilitated by the use of CRISPR/Cas9 [16] providing a marker-free genome editing tool, which can also be applied for engineering of industrial yeast strains [37, 46]. However, in vivo assembly is quite error-prone and hence extensive verification using PCR and sequencing must always be performed to check for the correct assembly. Moreover, to ensure the stability of large inserts, the multiple use of homologous sequences (promoters, terminators) should be avoided. Alternatively, yeast integrative vectors allow cloning of the genes and their subsequent integration into the genome [42, 45]. Such vectors enable a non-laborious errorless propagation of the cloned components and their reliable delivery into the cells without the need of laborious genotyping. The integrative vectors do not contain replication origin and they get integrated into a particular genomic location after their delivery into the cell via homologous recombination. To achieve reproducible levels of expression, it is important to select a suitable promoter for driving the expression of a gene [34] and an appropriate genomic location, since the chromatin structure can also influence heterologous gene expression. Traditionally, auxotrophic genes have acted as integration target sites [38, 47]. In addition, various transposable elements and other repetitive chromosomal locations were evaluated in terms of reliable expression [9, 10]. A series of vectors with various auxotrophic markers, promoters, multiple cloning sites and integration sequences have been developed [9, 15, 38, 47]. Recently, an EasyClone vector set was developed [17] enabling integration by double cross-over events into several intergenic regions, which have been validated for sufficient and reliable expression level and absence of growth impairment, on three different *S. cerevisiae* chromosomes [24]. The insertion sites are interspaced with essential genes voiding risk of chromosomal rearrangements even when the expression cassettes share long sequence homologies [26]. The EasyClone vectors enable cloning of one to two genes with the promoters of choice using convenient uracil-excision based (USER) cloning technique. The vectors offer several selection possibilities via auxotrophic markers and also enable marker recycling via Cre-loxP system [17]. However, they are not suitable for prototrophic strains.

In this work, we present the construction and evaluation of EasyClone 2.0 set of integrative vectors suitable for (over-)expression of (heterologous) genes in both laboratory and industrial *S. cerevisiae* strains. The vectors allow for selection in prototrophic yeast strains as they are combined with six different dominant selection markers. The vectors are based on the EasyClone system, which provides a possibility of cloning of up to two genes, when a bidirectional promoter is used [17] and integration of the construct into well-defined chromosomal locations [26]. We further show that the vector set enables stable integration of three different reporter genes, exhibiting a homogenous expression level in several unrelated industrial strains. The marker genes are flanked with loxP sites allowing for marker rescue and recycling, providing a possibility of repeated rounds of gene insertions. As a proof-of-concept, we introduced a seven-gene xylose consumption pathway into the industrial diploid strains Ethanol Red and CLIB382, the former being used in bioethanol production and the latter is applied as a brewing yeast.

## Materials and methods

### Strains and media

The following *S. cerevisiae* strains were included in this study: prototrophic haploid laboratory CEN. PK113-7D (obtained from Peter Kötter, Johann Wolfgang Goethe University Frankfurt, Germany), diploid industrial bioethanol producer Ethanol Red (obtained from Fermentis A Lesaffre division, France), bioethanol producer CBS7960, isolated from Brazilian ethanol fermentation plants, and an Irish brewer’s strain CLIB382 (both strains were obtained from Silas Villas-Bôas, University of Auckland, New Zealand). All the other strains are derivatives of the strains mentioned above and are listed in Table 1. Yeast cells were grown at 30 °C in standard yeast peptone dextrose (YPD) medium or synthetic complete (SC) medium supplemented with 20 g/l agar for preparation of solid medium. For selection, the media were supplemented with 200 mg/l G418 sulfate, 200 mg/l hygromycin B, 100 mg/l nourseothricin or 20 mg/l phleomycin. For selection on acetamide and d-serine, media containing 0.17 % yeast nitrogen base (without aminoacids and ammonium sulfate), 6.6 g/l of potassium sulfate and either 0.6 g/l acetamide or 2 g/l d-serine, respectively, were used. In particular cases in YPD medium, glucose was replaced by 20 g/l xylose (YPX) or 20 g/l galactose (YPGal). *E. coli* strain DH5α was used as a host for cloning and plasmid propagation. *E. coli* cells were grown at 37 °C in Luria–Bertani (LB) medium containing 100 mg/l ampicillin or 50 mg/l kanamycin.

**Table 1:** List of strains constructed in the study

Strain name	Parental strain	Description	Integrated vectors
CENstCFP	CEN.PK113-7D	X-2:TEF1p-CFP	pCfB2048
CENstRFP	CEN.PK113-7D	X-3:TEF1p-RFP	pCfB2049
CENstYFP	CEN.PK113-7D	X-4:TEF1p-YFP	pCfB2050
CENstFP1	CEN.PK113-7D	X-2:TEF1p-CFP X-3:TEF1p-RFP X-4:TEF1p-YFP	pCfB2048, pCfB2049, pCfB2050
CENstFP2	CEN.PK113-7D	X-2:TEF1p-CFP X-3:TEF1p-RFP X-4:TEF1p-YFP marker-less	pCfB2048, pCfB2049, pCfB2050
CENstGFP1	CEN.PK113-7D	X-2:TEF1p-GFP	pCfB3482
CENstGFP2	CEN.PK113-7D	X-3:TEF1p-GFP	pCfB3483
CENstGFP3	CEN.PK113-7D	X-4:TEF1p-GFP	pCfB3484
CENstGFP4	CEN.PK113-7D	XI-1:TEF1p-GFP	pCfB3485
CENstGFP5	CEN.PK113-7D	XI-2:TEF1p-GFP	pCfB3486
CENstGFP6	CEN.PK113-7D	XI-3:TEF1p-GFP	pCfB3487
CENstGFP7	CEN.PK113-7D	XI-5:TEF1p-GFP	pCfB3488
CENstGFP8	CEN.PK113-7D	XII-1:TEF1p-GFP	pCfB3489
CENstGFP9	CEN.PK113-7D	XII-2:TEF1p-GFP	pCfB3490
CENstGFP10	CEN.PK113-7D	XII-4:TEF1p-GFP	pCfB3491
CENstGFP11	CEN.PK113-7D	XII-5:TEF1p-GFP	pCfB3492
ERstCFP	Ethanol Red	X-2:TEF1p-CFP	pCfB2048
ERstRFP	Ethanol Red	X-3:TEF1p-RFP	pCfB2049
ERstYFP	Ethanol Red	X-4:TEF1p-YFP	pCfB2050
ERstFP1	Ethanol Red	X-2:TEF1p-CFP XI-1:TEF1p-YFP XII-4:TEF1p-RFP	pCfB2048, pCfB2515, pCfB2516
ERstFP2	Ethanol Red	X-2:TEF1p-CFP XI-1:TEF1p-YFP XII-4:TEF1p-RFP marker- less	pCfB2048, pCfB2515, pCfB2516
ERstFP3	Ethanol Red	X-2:TEF1p-CFP X-3:TEF1p-RFP X-4:TEF1p-YFP	pCfB2048, pCfB2049, pCfB2050
CBSstFP1	CBS7960	X-2:TEF1p-CFP XI-1:TEF1p-YFP XII-4:TEF1p-RFP	pCfB2048, pCfB2515, pCfB2516
CLIBstFP1	CLIB382	X-2:TEF1p-CFP XI-1:TEF1p-YFP XII-4:TEF1p-RFP	pCfB2048, pCfB2515, pCfB2516
CENstXYL	CEN.PK113-7D	δTy2:TEF1p-CpXylA/TDH3p-PsXYL3 X-2:TEF1p-RPE1/TDH3p-RKI1 XI-5:TEF1p-PsTAL1/TDH3p-TKL1 XII-2:TEF1p-CpXylA/TDH3p-PsSUT1	pCfB2871, pCfB2523, pCfB2872, pCfB2874
ERstXYL	Ethanol Red	δTy2:TEF1p-CpXylA/TDH3p-PsXYL3 X-2:TEF1p-RPE1/TDH3p-RKI1 XI-5:TEF1p-PsTAL1/TDH3p-TKL1 XII-2:TEF1p-CpXylA/TDH3p-PsSUT1	pCfB2871, pCfB2523, pCfB2872, pCfB2874
CLIBstXYL	CLIB382	δTy2:TEF1p-CpXylA/TDH3p-PsXYL3 X-2:TEF1p-RPE1/TDH3p-RKI1 XI-5:TEF1p-PsTAL1/TDH3p-TKL1 XII-2:TEF1p-CpXylA/TDH3p-PsSUT1	pCfB2871, pCfB2523, pCfB2872, pCfB2874

### Plasmid construction

**Fig. 1:**
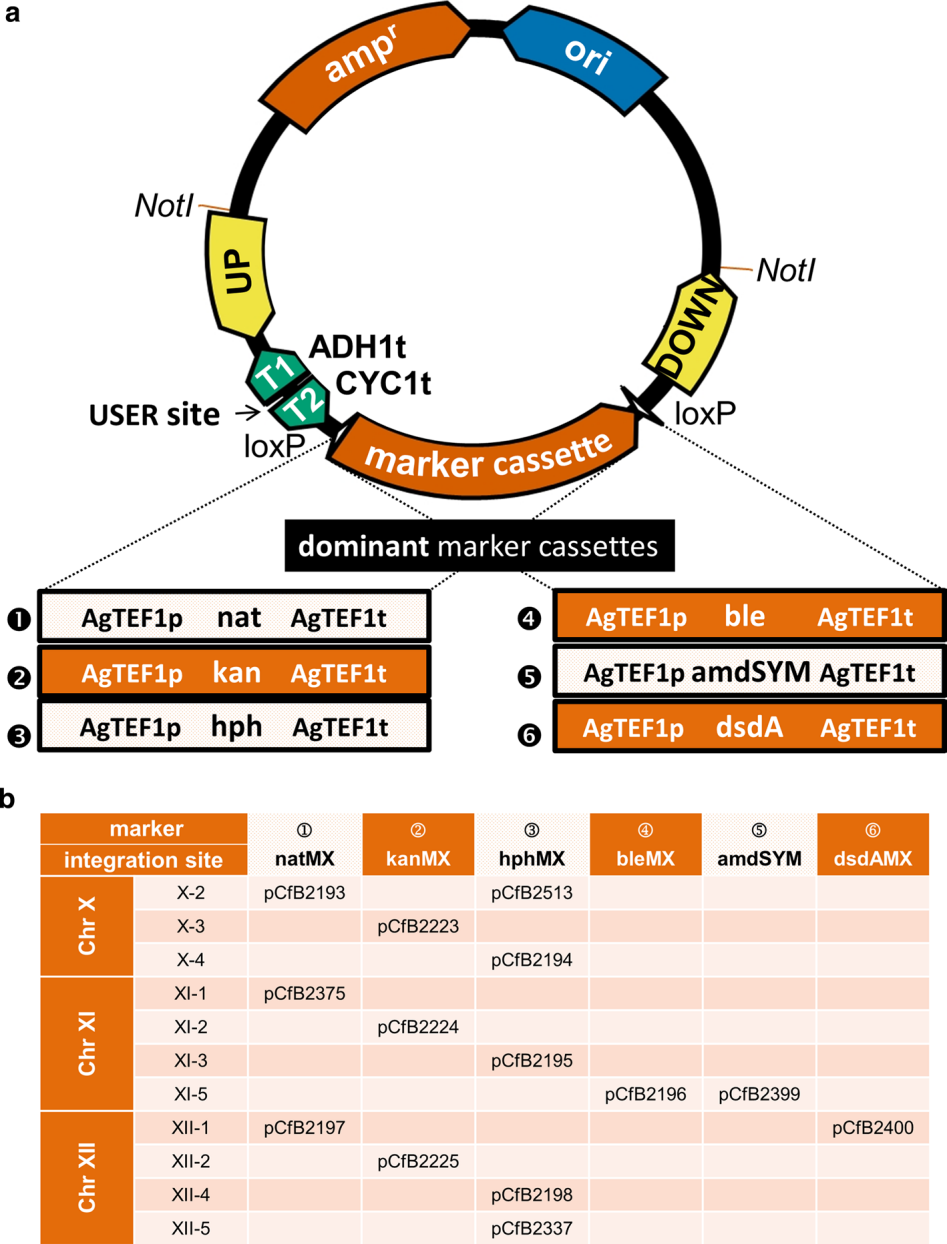
New set of EasyClone2.0 vectors with dominant markers. **a** Schematic illustration of the vector structure and the dominant marker cassettes. **b** Table of the vectors presenting combinations of particular vector (integration site) with the dominant markers

The new set of integrative vectors (Fig. 1, Supplementary material Table S1, the vector set along with maps and sequences can be obtained from Addgene) was constructed by USER fusion [31]. The particular BioBricks (original vector backbone, the details on construction are provided in Supplementary material Supplementary Methods, and marker gene expression cassettes) (Supplementary material Table S2) were amplified by PCR with PfuX7 polymerase [30] under the following conditions: 98 °C for 2 min, 30 cycles of 98 °C for 10 s, 54 °C for 10 s, 72 °C for 30 s/1 kb, 72 °C for 10 min. The used templates and primers are listed in Supplementary material Table S1 and Table S3. All the marker gene cassettes (Fig. 1, Supplementary material Table S2) were synthesized by GeneArt (Life Technologies). DNA fragments were gel purified and incubated in HF buffer (New England BioLabs) together with USER enzyme (New England BioLabs) for 25 min at 37 °C followed by incubation at 25 °C for 25 min. The reactions were transformed into chemically competent *E. coli* cells. For cloning of particular genes, the vectors were digested with FastDigest *AsiSI* (Life Technologies) restriction endonuclease, nicked with *Nb.BsmI* (New England BioLabs) and assembled with PCR amplified genes and promoter(s) of choice by USER reaction and subsequent transformation into *E. coli*. Heterologous genes were either synthesized by GeneArt or amplified from the genomic DNA of *S. cerevisiae* CEN.PK113-7D or *Pichia stipitis* DSM-3651 (obtained from The Leibniz Institute DSMZ—German Collection of Microorganisms and Cell Cultures) (Supplementary material Table S2). To test the selection properties of the dominant markers, the disruption cassettes for homologous recombination-mediated gene replacement were prepared by PCR [36]. One cassette consisted of the upstream *URA3* region and the first 2/3 part of a particular marker, and the other cassette contained the last 2/3 part of the marker and the downstream sequence of *URA3*. Both fragments were made by fusion PCR and used for yeast transformation.

### Strain construction

Before yeast transformation, the integrative vectors were linearized by FastDigest *NotI* (Life Technologies) restriction enzyme. Yeast cells were transformed by PEG/LiAc method according to [12]. The heat shock time was prolonged to 90 min for strain CBS7960 giving on average 3-fold higher transformation efficiency in this particular strain. Marker loop-out was performed by transforming the cells with Cre-recombinase carrying centromeric vector (Supplementary material Table S1). The Cre-recombinase expression was induced by cultivation of the yeast cells in YPGal medium for 6 h. After the induction, the cells were plated on non-selective YPD plates. After 2 days, the colonies were replica-plated on selective media containing particular selection agent. The clones lacking the selection marker(s) were verified by colony PCR.

### Stability of reporter gene integration

Yeast strains expressing three reporter genes were grown in three replicates in 1 ml of non-selective YPD medium in deep-well plates at 30 °C with agitation at 300 rpm. Approximately, every 20 h 50 μl of the culture was transferred into fresh medium. Four serial transfer cycles were performed for a total of approximately 35 generations. The final strains along with the parental strains were analyzed by flow cytometry.

### Sensitivity of the strains to selection agents

The strain sensitivity was tested by the drop assay. The cells were pre-grown in YPD overnight. Subsequently, a water suspension containing 10^8^ cells per ml was prepared. Then, 5 μl drops of 10-fold serial dilutions were applied to YPD plates supplemented with particular antibiotic. Plates were scored after 2 days of growth.

### Fluorescence microscopy and fluorescence measurement

The fluorescent protein expressing cells were observed under 100×/1.25 immersion oil objective using Leica DM4000 fluorescence microscope. The pictures were captured using Leica DFC300FX digital color camera and LAS 4.0 software. Excitation BP436/12, BP500/20, BP545/40 and suppression filters BP467/37, BP535/30, BP610/75 were used for CFP, YFP and RFP, respectively. Measurement of GFP fluorescence was carried out in standard round 96-well microtiter plates with an optical bottom (Greiner Bio-one, Germany). The fluorescence levels were monitored using Biotek Synergy MX multi-mode plate reader in 485/520 nm emission/excitation wavelength. The sensitivity of the photomultiplier was adjusted to 80 %. Overnight cultures of selected clones were twenty times diluted into SC media (with complete supplement mixture) and grown for 6 h with shaking. GFP fluorescence and OD_600nm_ of ten times diluted samples were determined.

### Flow cytometry

The cells grown overnight in YPD medium were washed twice with water, resuspended in phosphate-buffered saline buffer and subsequently analyzed on a BD FACSAriaflow cytometer equipped with three solid-state diode lasers: air-cooled Coherent™ Sapphirelaser (488 nm, 100 mW), Yellow Green laser (561 nm, 100 mW) and Deep Blue laser (445 nm, 50 mW). The following filters were used: FITC-A, PECy5-A, and mCFP-A for the analysis of emission of yellow fluorescent protein (YFP), red fluorescent protein (RFP), and cyan fluorescent protein (CFP), respectively. Compensation was performed according to the BD FACSDiva software protocol. Flow cytometry data sets were subsequently analyzed and interpreted by FlowJo software.

### HPLC analysis

The engineered xylose consuming strains were cultivated in three replicates in 50 ml of YPX medium in shake flasks with constant agitation (250 rpm). Samples were collected at certain time points and the concentration of xylose in culture supernatants was determined by HPLC (UltiMate 3000, Dionex). The samples were analyzed for 30 min using Aminex HPX-87H ion exclusion column with a 5 mM H_2_SO_4_ flow of 0.6 ml/min. The injection volume was 30 μl. The temperature of the column was 60 °C. Xylose was detected using RI-101 Refractive Index Detector (Dionex). The refractive index was measured at 45 °C. The data were acquired and analyzed with Chromeleon software.

### Quantitative PCR (qPCR) analysis of gene copy number

For determination of *CpXylA* gene copy number, qPCR analysis was performed using *ALG9* as a reference gene. Primers (Supplementary material Table S3) were designed using the PrimerQuest design tool (Integrated DNA Technologies). DNA extraction was carried out using ZR Fungal/Bacterial DNA MicroPrep™ Kit (Zymo Research). The qPCRs run in three replicates were performed on the Mx3000P qPCR System (Agilent Technologies) using Brilliant III Ultra-Fast QRT-PCR Master Mix (Agilent Technologies), forward and reverse primers (final concentration 200 nM), genomic DNA (0.001–50 ng per 20 μl reaction) from strains CENstXYL, ERstXYL and CLIBstXYL (or water in negative controls) and 0.3 μl per 20 μl reaction of 1:500 diluted reference dye. An initial melting/activation step at 95 °C for 10 min was followed by 40 cycles of melting and annealing/extension (95 °C for 20 s, 60 °C for 22 s) with a fluorescence measurement at the end of each amplification step. Raw data were analyzed using MxPro software (Agilent Technologies). PCR efficiency calculated from the slope of the standard curve was 90 %. Ct values of 5 ng dilutions of genomic DNA (providing standard deviation lower than 0.23) were used for determination of a gene copy number. The normalized gene copy number was calculated by absolute quantification based on standard curve of the reference gene (assuming 1 copy of the *ALG9* per haploid genome) using the following equation: ${\text{copy number}} (CpXylA) = \frac{{{\text{copy quantity}} (CpXylA)}}{{{\text{copy quantity }}(ALG9)}}.$

## Results

### Construction of a set of EasyClone vectors with dominant selection markers

To enable the use of the EasyClone system [17] for engineering of industrial yeast strains, we constructed a new set of integrative vectors allowing for selection of a genetic intervention in prototrophic strains (Fig. 1a). For such vectors, an auxotrophic selection cassette in the original set was substituted for one of the six synthetic dominant selection markers (Fig. 1a). Selection genes conferring resistance to several substances (with resistance to antibiotics as the most common) are available. Initially, CEN.PK, Ethanol Red, CBS7960 and CLIB382 strains were tested for their sensitivity to antibiotics G418, nourseothricin, hygromycin B and phleomycin. All the strains grown on YPD were sensitive to the antibiotics in common concentrations as follows: G418—200 mg/l, nourseothricin—100 mg/l, hygromycin B—200 mg/l and phleomycin—20 mg/l (Supplementary material Fig. S1). Two other dominant marker gene expression cassettes were used. This involved amdSYM cassette containing acetamidase gene which confers yeast cells the ability to use acetamide as a nitrogen source [43] and dsdAMX containing d-serine deaminase gene which confers yeast cells resistance to d-serine and the ability to use d-serine as a nitrogen source [50]. The ability of all marker gene expression cassettes (containing marker gene, *TEF1* promoter and *TEF1* terminator from *Ashbya gossypii*) to confer the resistance was evaluated. Yeast cells were transformed with PCR-generated disruption cassettes carrying the marker genes and selected on corresponding media. Randomly selected transformants were verified for presence of the particular marker cassette. An appearance of neither false positives nor spontaneous resistance mutants was observed. Certain background growth on media containing acetamide or d-serine appeared. However, the positive transformants always got a growth advantage and significantly overgrew the background (not shown).

### EasyClone2.0 vectors allow stable integration of multiple genes into industrial strains

**Fig. 2:**
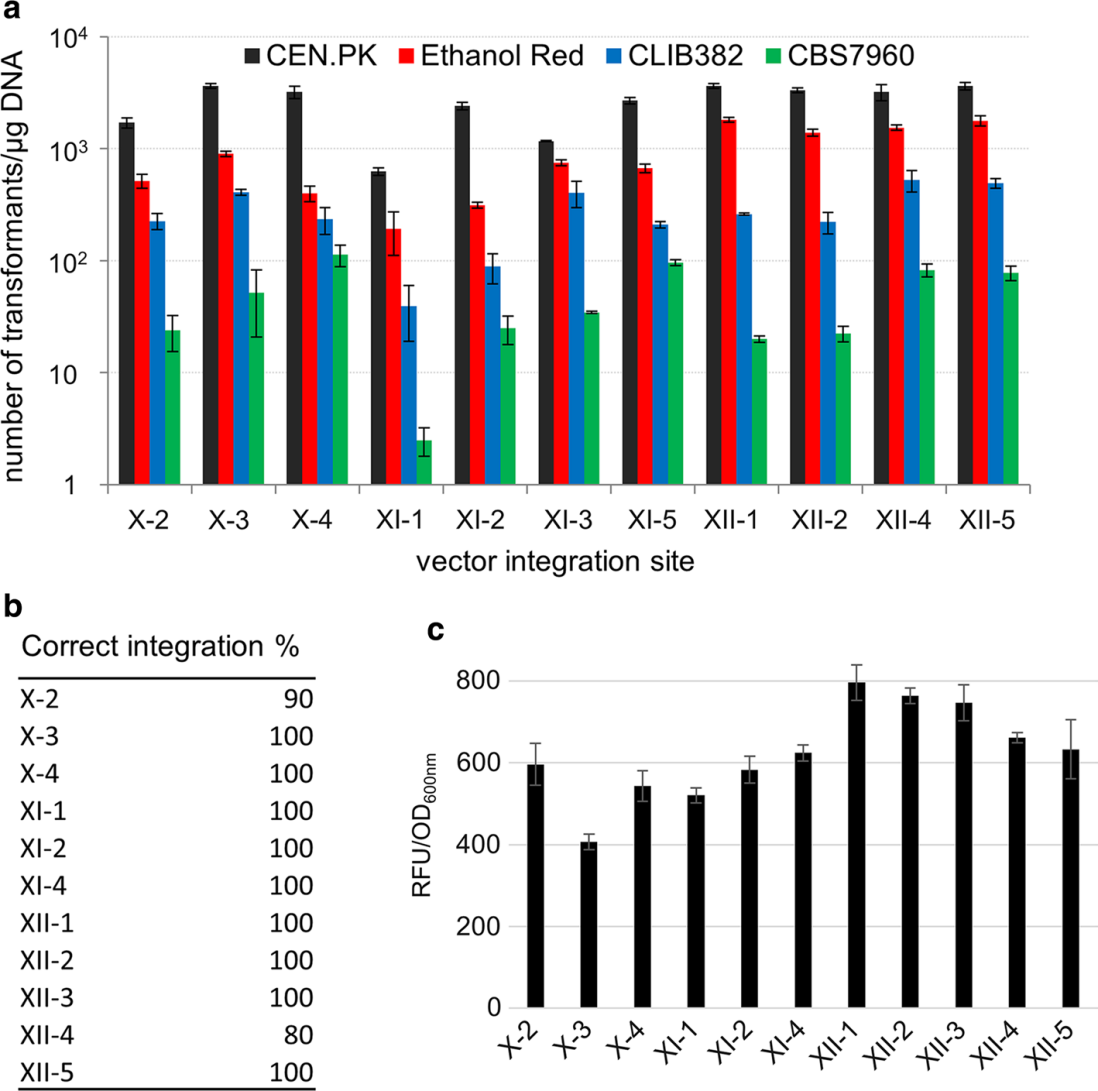
Characteristics of the EasyClone 2.0 vectors. **a** Transformation efficiency of CEN.PK, Ethanol Red, CLIB382 and CBS7960 strains with the EasyClone 2.0 vectors. Values of the transformation efficiency (expressed as a number of transformants per μg DNA) are plotted on logarithmic scale. The values represent an average from two parallel experiments. *Error bars* represent standard deviation (*N* = 2). The amount of 10^8^ cells was used for each strain and plasmid transformation. **b** Percentage of correct vector integration as evaluated by colony PCR of ten randomly selected CEN.PK clones. **c** Relative fluorescence intensity of ten randomly selected CEN.PK clones. *Error bars* represent standard deviation (*N* = 10)

The vectors carrying all eleven insertion sequences (Fig. 1b) were examined and mutually compared in terms of transformation efficiency, correct integration and reporter expression level. To evaluate this, GFP reporter gene under control of constitutive *TEF1* promoter was cloned into the vectors carrying dominant markers and the resulting vectors (Supplementary material Table S1) were subsequently transformed into the CEN.PK, Ethanol Red, CLIB382 and CBS7960 cells. The reporter gene carrying vectors were delivered into the CEN.PK strain with comparable transformation efficiency in a range of 1.2 × 10^3^– 3.7 × 10^3^ cells/μg DNA, with an exception of XI-1 insertion sequence-containing vector that yielded lower amount (6.25 × 10^2^) of transformants (Fig. 2a). The efficiency was not surprisingly lower in case of the industrial strains and differed more among particular vectors being in a range of 1.9 × 10^2^ − 1.8 × 10^3^ cells/μg DNA in case of Ethanol Red strain, 40–5.3 × 10^2^ cells/μg DNAin CLIB382 and 2–1.13 × 10^2^ cells/μg DNA in CBS7960 strain, with the vector XI-1 providing the lowest yield in all cases (Fig. 2a). The vectors integrated at the desired insertion site in the genome of the CEN.PK strain with 80–100 % accuracy (Fig. 2b), as verified by evaluation of randomly selected clones by colony PCR. GFP fluorescent levels were examined under the bench top UV transilluminator revealing homogenous level of the fluorescence. Fluorescence of ten clones of the CEN.PK strain from each vector transformation was determined in microtiter plates by the fluorescence plate reader. The results showed homogenous level of expression among clones from the particular insertion site integration. The specific fluorescence values were comparable between the vectors, though some integration sites gave slightly lower values (insertion site X-2) and some slightly higher (insertion site XII-1) (Fig. 2c). This demonstrates the sufficient, homogenous and reliable level of expression, which the integration into these chromosomal locations mediated by the new vector set ensures.

**Fig. 3:**
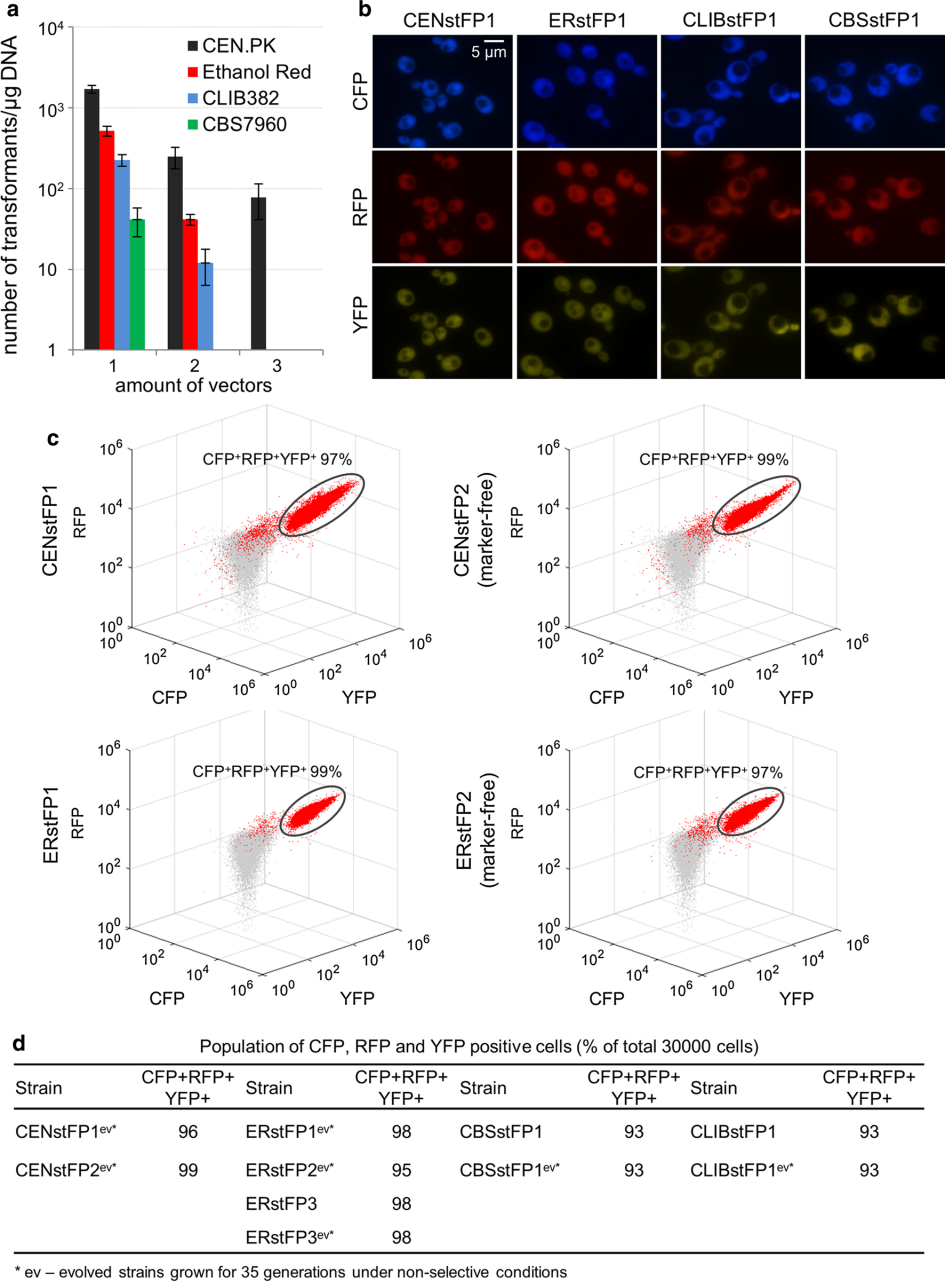
Integration of reporter genes delivered by the EasyClone 2.0 vectors in industrial yeast. **a** Transformation efficiency of the strains when transformed with 1, 2 or 3 vectors simultaneously. Values of the transformation efficiency (expressed as a number of transformants per μg DNA) are plotted on logarithmic scale. The values represent an average from two independent experiments. *Error bars* represent standard deviation (*N* = 2). The amount of 10^8^ cells was used for each transformation. **b** Cells of the laboratory (CENstFP1) and industrial (ERstFP1, CBSstFP1, CLIBstFP1) strains expressing three different fluorescent proteins—CFP, RFP, YFP. **c** Flow cytometry analysis of the fluorescent protein expressing strains CENstFP1 (marker-containing), CENstFP2 (marker-free), ERstFP1 (marker-containing), ERstFP2 (marker-free). Three-dimensional scatter plots demonstrate the population properties of the fluorescent protein-expressing strains (*red* *populations*). The values were plotted using scatter 3 function in MATLAB. Wild type (fluorescence negative) control overlay (*grey populations*) is given for comparison. The percentages of CFP^+^YFP^+^RFP^+^ populations (delineated by the ellipse), as obtained by sequential gating in FlowJo (Supplementary material Fig. S2), are given. Two-dimensional contour plots of such populations are shown in Supplementary material Fig. S2. **d** The *table* displays the percentages of CFP^+^YFP^+^RFP^+^ populations of the reporter gene expressing strains and their derivatives grown for 35 generations under non-selective conditions. The populations were analyzed in FlowJo as described in Supplementary material Fig. S2. Two-dimensional contour plots of such populations are shown in Supplementary material Fig. S2

To evaluate integration of prepared vectors and expression of cloned gene(s) in different industrial strains, three reporter genes encoding fluorescent proteins CFP, RFP and YFP under control of *TEF1* promoter were cloned into the selected EasyClone vectors carrying different dominant markers, targeting insertion sites located at the same chromosome as well as the sites located at three different chromosomes (X, XI, XII) (Supplementary material Table S1). Such vectors were subsequently transformed into the laboratory CEN.PK and industrial Ethanol Red, CBS7960 and CLIB382 strains. All vectors could be introduced simultaneously into the CEN.PK strain, while in Ethanol Red and CLIB382 strains just two vector transformation yielded successful transformants. A second transformation was performed to introduce the third vector. In case of CBS7960 strain, reporter genes carrying vectors had to be introduced sequentially (Fig. 3a). As shown by fluorescence microscopy, the cells of each strain indeed expressed all three fluorescent proteins at homogenous level (Fig. 3b), independent of the combination of the vectors used (not shown).

### Selection markers can be removed by Cre-loxP recombination

All the selection markers are flanked by loxP sites (Fig. 1a) allowing marker rescue mediated by CreA site-specific recombinase [13]. A one-step marker loop-out was performed in all tested strains by transforming the cells with a Cre-recombinase containing plasmid and subsequent induction of Cre expression. The insertion sites are interspaced by essential genetic elements, which prevents loop-out of the inserted genes in haploid laboratory strains [26]. This advantage of the platform is naturally lost when used in di-/polyploid yeast. Therefore, there is a risk of inserted fragment loop-out caused by Cre-recombinase when neighboring insertion sites are used, encountering relatively close distance of several loxP sites. Indeed, a loss of at least one of the inserted reporter genes could be observed after Cre-mediated marker loop-out in case of industrial strains carrying the reporter genes on the same chromosome, while expectedly, there was no loss of the inserted genes in haploid CEN.PK strain. However, when the reporter genes were inserted at different chromosomal locations, a one-step marker removal was possible in diploid Ethanol Red strain without loss of the inserted genes. Flow cytometer analysis of the cell population did not reveal any significant changes of the populations in terms of the reporter gene fluorescence when comparing marker-containing and marker-free strains (Fig. 3c). To test the stability of the inserted genes, all reporter gene containing strains were grown for 35 generations under non-selective conditions. The resulting populations (including also several variants of industrial Ethanol Red, i.e., with the reporters on single chromosome, with the reporters on different chromosomes, both marker-containing and marker-free) were analyzed by flow cytometry. This analysis did not reveal any loss of the reporter genes in any strain or other significant differences between parental and derived strains. In CEN.PK and Ethanol Red strain derivatives, the vast majority of cells (more than 95 %) exhibited a homogenous level of reporter gene fluorescence (Fig. 3d, Supplementary material Fig. S2). Only a small part of the cell population of industrial CBS7960 and CLIB382 derivatives (around 7 %) showed decrease in fluorescence of the reporters. However, this was characteristic also for the parental population and not affected by the prolonged growth in selection-free conditions (Fig. 3d, Supplementary material Fig. S2). This demonstrated that the constructed EasyClone 2.0 vector set is suitable for stable heterologous gene insertions in industrial yeast and that dominant markers can be recycled using the Cre/loxP system.

### Construction of xylose utilizing strains

**Fig. 4:**
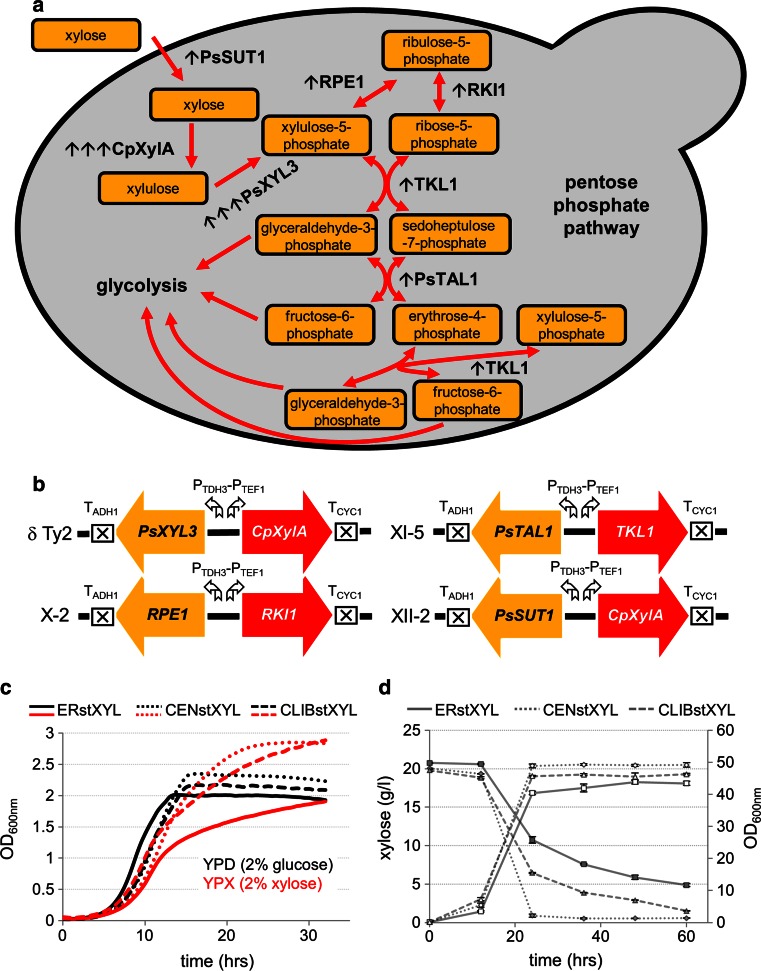
Engineering of industrial strains for xylose utilization. **a** Schematic illustration of the xylose isomerase pathway. The genes integrated using the EasyClone2.0 vectors are displayed. *Single black arrows* (↑) represent single integration*, triple arrows* (↑↑↑) represent multiple integration. **b** Schematic illustration of chromosomal insertions of the cloned xylose pathway genes in the genomes of CENstXYL, ERstXYL and CLIBstXYL strains. Particular chromosomal locations are displayed as well as promoters and terminators. **c** Growth curves of CENstXYL, ERstXYL and CLIBstXYL strains. The strains were grown in 100 μl of YPD (*black* *curves*) and YPX (*red curves*) media in microtiter plates, OD was determined with Biotek ELx808 microplate reader in 30 min intervals. The values were normalized to a 1 cm pathlength using pathlength correction performed according to manufacturer’s instructions. **d** The graph represents time course of xylose consumption by CENstXYL, ERstXYL and CLIBstXYL strains grown in 50 ml YPX medium in shake flasks. Samples were taken at regular time points and OD of the culture was determined with the NanoPhotometer Pearl (Implen, Germany) in a 1 cm pathlength cuvette, and xylose concentration was measured by HPLC. Xylose concentration is plotted on the primary *y* *axis* (*closed symbols*), OD values (*open symbols*) on the secondary *y axis*. The cultures were cultivated in triplicates. *Error bars* represent standard deviation (*N* = 3)

The development of industrial xylose-fermenting yeast is of a great importance for second and third-generation biorefineries utilizing lignocellulosic feedstocks [22]. Two pathways for xylose utilization that have been mostly studied are oxidoreductase pathway, involving the expression of xylose reductase and xylitol dehydrogenase [18], and the xylose isomerase pathway, involving expression of heterologous xylose isomerase enzyme and xylulokinase [5]. The latter pathway does not have a cofactor imbalance issue and provides higher yields under industrially relevant anaerobic conditions [6, 21]. We decided to construct xylose consuming strains with different industrial backgrounds as a case study, demonstrating the use of our integrative vector toolbox. To perform this, a strategy using the introduction of the xylose isomerase pathway and tuning of pentose phosphate pathway was selected (Fig. 4a). The crucial step is to ensure sufficient activity of heterologous xylose isomerase. To be able to introduce more copies of a gene, we constructed multiple integration vectors (Maury et al., unpublished results), targeting LTR sequences of yeast Ty2 and Ty4 transposable elements spread in yeast genome in several copies, allowing for selection in prototrophic yeast (Supplementary material Table S1). The vector with Ty2 integration site was used for cloning of the *XylA* gene from *Clostridium phytofermentans* [5] along with the *P. stipitis XYL3* gene encoding d-xylulokinase. Selected single integration vectors were used for cloning of the *P. stipitis SUT1* gene (along with a *CpXylA* allele), encoding a sugar transporter with higher affinity for xylose, and 4 genes of the non-oxidative part of the pentose phosphate pathway (PPP): three native *S. cerevisiae* genes (*RKI1*, *RPE1*, *TKL1*) and transaldolase from *P. stipitis* (*PsTAL1*) (Fig. 4b). We chose the *PsTAL1* gene because it has been shown previously that its overexpression does not influence the growth rate, in contrast to the native *TAL1* allele [19]. The genes were cloned under control of the strong constitutive promoters *P*_*TEF1*_ and *P*_*TDH3*_ (Fig. 4b). The vectors were successfully inserted into the genomes of laboratory CEN.PK and industrial Ethanol Red and CLIB382 strains, chosen to represent two different industrial backgrounds. The insertion of three vectors was achieved in a single transformation for laboratory strain and in two transformation rounds for industrial strains. Finally, the resulting strains were transformed with Ty2 vector carrying *CpXylA* and *PsXYL3* genes. To select for multiple integration, the concentration of selective antibiotic was increased 5-fold, and xylose containing complex medium was used. The resulting transformants were screened for growth in YPX (and YPD for comparison) liquid medium using microplate reader. The fastest growing isolates (Fig. 4c) were evaluated in time course experiment in shake flasks. Furthermore, *CpXylA* gene copy number of such strains was determined by qPCR. CENstXYL (containing 6 copies of *CpXylA* gene), ERstXYL (8 copies of *CpXylA*) and CLIBstXYL (8 copies of *CpXylA*) strains were grown for 60 h under aerobic conditions in complex YPX medium and the xylose concentration and the maximum specific growth rates *μ*_max_ were determined. CENstXYL strain exhibited the fastest growth (*μ*_max_ = 0.296 h^−1^) and reached the highest OD (approximately after 24 h of growth), CLIBstXYL strain showed similar parameters (*μ*_max_ = 0.283 h^−1^) though reaching slightly lower OD, while ERstXYL strain grew slower (*μ*_max_ = 0.271 h^−1^, Supplementary material Fig. S3). CENstXYL strains consumed the entire xylose content in 24 h, and CLIBstXYL strains in 60 h. In ERstXYL cultivation, 75 % of the xylose content was consumed after 60 h (Fig. 4d).

## Discussion

Stress-tolerant and robust industrial yeast strains are suitable production organisms in large-scale industrial biorefineries, where biomass feedstocks are fermented into fuels and chemicals [32]. Heterologous gene insertions and their controlled expression are crucial events needed for the introduction of new cellular processes enabling production of non-natural products and optimization of the desired cellular properties. A common feature for genetic manipulations is the requirement of genetic markers that enable the selection of mutants [25]. Engineering of prototrophic industrial yeast strains prevents the use of auxotrophic selection and requires use of dominant markers [2]. The presented EasyClone 2.0 set of integrative vectors allow for gene insertions and selection in prototrophic yeast strains. The substances used as selection agents do not show any cross-reactivity and can be used as selective agents simultaneously. This is especially important when more genes are considered to be introduced. Previously presented vector sets with dominant markers do not provide such broad option of marker selection and/or provide only limited options of promoter choice to regulate the expression of an inserted gene [23, 47]. The EasyClone vectors enable free choice of any promoter to control the expression [17]. Level of expression, its homogeneity and reliability are an important consideration of any industrial process design. This is an issue when episomal vectors are used, since the homogenous expression cannot be controlled [17, 42]. Although possible to use in prototrophic strains, e.g., for a gene expression evaluation [49], such vectors are not suitable for construction of efficient cell factories. The constructed second generation of EasyClone vectors, designed to integrate into previously validated integration site on three different *S. cerevisiae* chromosomes [26], provides sufficient and mutually comparable level of expression.

Industrial strains often display low accessibility in terms of foreign DNA take up [44]. The vector set is equipped with long insertion sequences. This provides universality of such vectors as shown by the successful insertion and expression of three different reporter genes in unrelated strains, despite originally designed for laboratory strains [26]. This is an apparent advantage when compared to previously constructed vector sets integrating at also well-defined chromosomal locations, using however PCR-generated short insertion sequences [9]. Short homologous sequences might cause lower probability of successful insertion and subsequent lower yield of correct transformants. Furthermore, sufficiently long insertion regions allowed introduction of several vectors simultaneously in the tested strains, except the CBS7960, which represents a hardly accessible strain in general [46]. Besides the homogenous and controlled level of expression, the stability of the inserted genetic material is another important consideration. Diploid industrial strains lack the advantage of the expression platform interspaced by essential genes, preventing homologous recombination-mediated gene loss [26]. Despite this, the presented set enables stable integration expression of heterologous genes after prolonged cultivation under non-selective conditions. Even when genes are inserted on the same chromosome, no loss of the inserted reporters was observed.

Some previously reported dominant marker containing vectors lack marker excision possibility and thus prevent repeated use of the vectors and markers [47]. Moreover, the final production strains should not contain antibiotic resistance markers to avoid rise of antibiotics resistance among pathogens via horizontal gene transfer. The presented set carry markers flanked with loxP sites allowing for one-step marker rescue [13], being widely used marker recycling strategy [40]. The successful one-step marker excision was demonstrated. However, when the genes were inserted on the same chromosome, Cre-mediated marker loop-out was accompanied also by the loss of a reporter gene in diploid strains. This happened most likely due to accumulation of six loxP sites in relatively short, 42 kbp, distance [17]. Thus, one must pay special attention to a selection of suitable vectors and planning of simultaneous and sequential integrations to avoid such situation when excision of the markers in diploid strains is intended.

For the development of third-generation biorefineries, producing valuable chemicals from non-food biomass such as lignocellulosic substrates, conversion of C5-rich streams is an important cost-reductive challenge. For this reason, many studies have faced this challenge and succeeded in engineering of even industrial *S. cerevisiae* strains, naturally not fermenting C5 streams, for utilization of C5 sugars [37–39]. Based on great importance of C5-fermentation in *S. cerevisiae*, we constructed xylose consuming strains as a valuable proof-of-concept of applicability of the constructed vector set in metabolic engineering of industrial strains. To ensure sufficiently high expression (activity) of xylose isomerase, we enriched our vector set for vectors targeting δ sequences of Ty2 transposons (Maury et al., unpublished results). These elements are spread in several copies in yeast genome and enable insertion of a gene in many copies [39]. In contrast to the stable nature of single-site genome integrations, certain stability issues of multiple copy δ-based insertions have been documented [51]. However, our strategy considered the presence of xylose acting as natural selection pressure, preventing the potential risk of xylose isomerase gene loss. Moreover, the use of such insertion sites might facilitate further multiplication of *XylA* copy number, an event responsible for improved xylose metabolism after long-term evolution in the presence of xylose [7]. The engineered strains were able to utilize xylose as sole carbon source, with different properties dependent apparently on strain background. Further optimizing strategies, e.g., knock-out of *GRE3* to avoid production of xylitol as an inhibitor of xylose isomerase activity [21] and adaptive laboratory evolution [6] will be pursued in the future work to improve the xylose metabolism.

Taken together, we constructed the second generation of integrative vectors which mediate (heterologous) gene expression in industrial yeast (overview of EasyClone 2.0 in Supplementary material Fig. S4). The vectors provide (1) a wide range of dominant marker options for selection in (prototrophic) yeast strains, (2) free choice of a promoter and homogenous level of expression, (3) universality for use in strains with different genetic background, (4) stability of the inserted genes and (5) marker excision and recycling possibilities for repeated rounds of gene insertions. Our results also document the applicability of the vector set for metabolic engineering of industrial strains. The presented toolbox significantly broadens the options of design and engineering of industrial cell factories.

## Electronic supplementary material

Supplementary material 1 (pptx 2743 kb)

Supplementary material 1 (docx 59 kb)
