# Supplementary material for: EasyClone 2.0: expanded toolkit of integrative vectors for stable gene expression in industrial Saccharomyces cerevisiae strains
Source: J Ind Microbiol Biotechnol. 2015 Sep 16;42(11):1519–31. doi: 10.1007/s10295-015-1684-8 (PMC4607720; doi:10.1007/s10295-015-1684-8)
Supplement: Supplementary file 1 — Supplementary material 1 (pptx 2743 kb) [file 10295_2015_1684_MOESM1_ESM.pptx]

## Slide 1
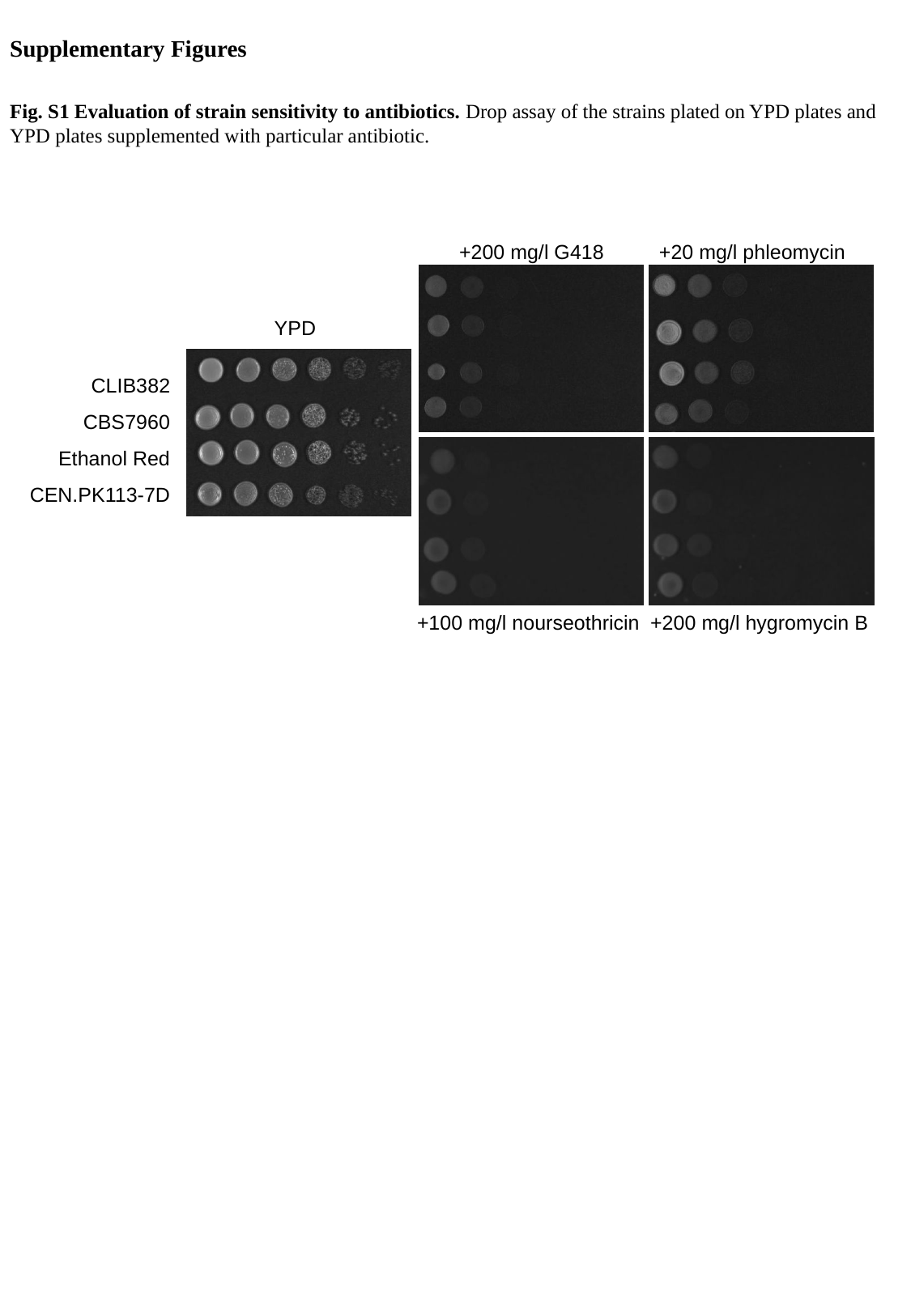

Supplementary Figures
Fig. S1 Evaluation of strain sensitivity to antibiotics. Drop assay of the strains plated on YPD plates and YPD plates supplemented with particular antibiotic.
+200 mg/l G418
+20 mg/l phleomycin
YPD
CLIB382
CBS7960
Ethanol Red
CEN.PK113-7D
+100 mg/l nourseothricin
+200 mg/l hygromycin B

## Slide 2
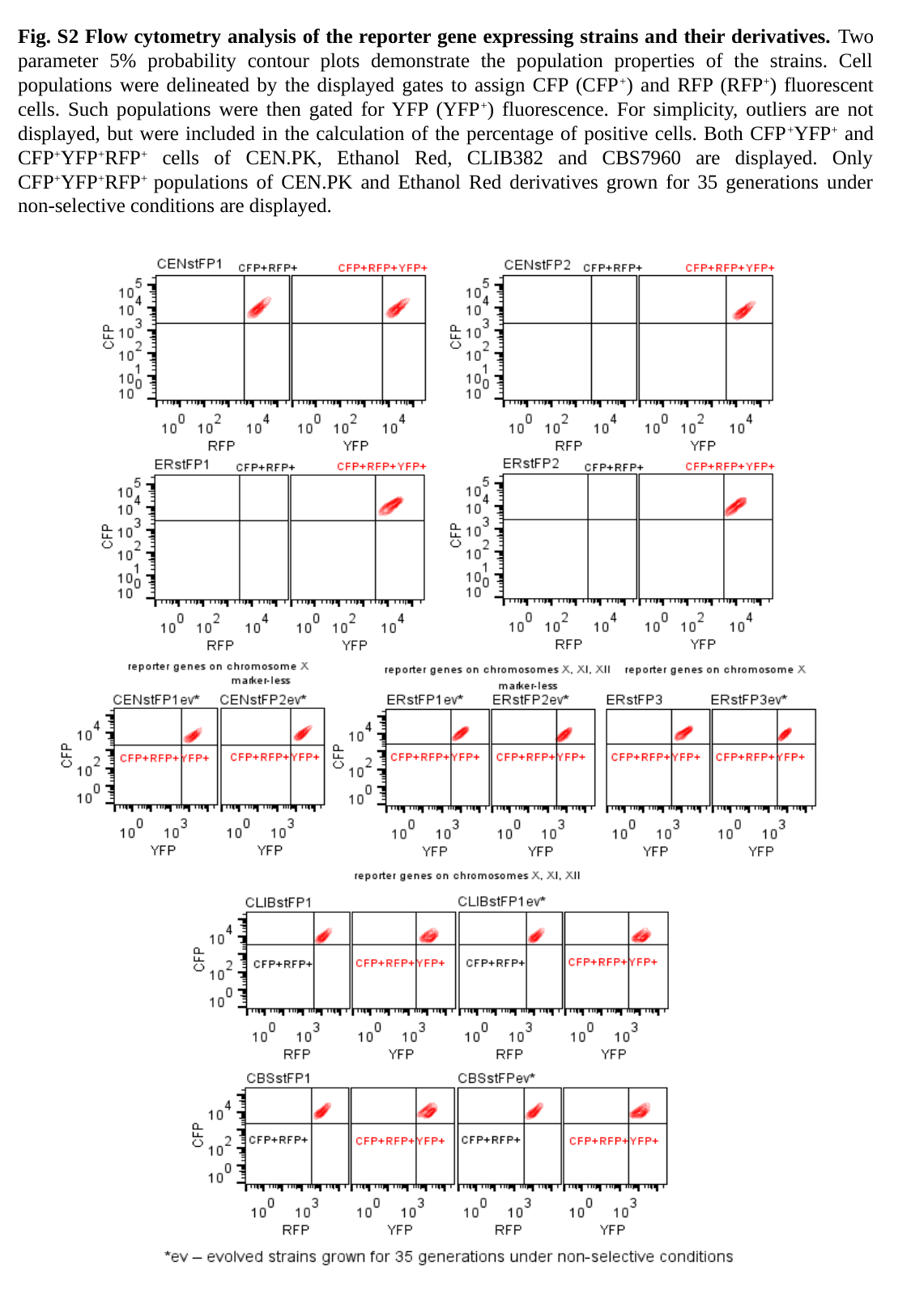

Fig. S2 Flow cytometry analysis of the reporter gene expressing strains and their derivatives. Two parameter 5% probability contour plots demonstrate the population properties of the strains. Cell populations were delineated by the displayed gates to assign CFP (CFP+) and RFP (RFP+) fluorescent cells. Such populations were then gated for YFP (YFP+) fluorescence. For simplicity, outliers are not displayed, but were included in the calculation of the percentage of positive cells. Both CFP+YFP+ and CFP+YFP+RFP+ cells of CEN.PK, Ethanol Red, CLIB382 and CBS7960 are displayed. Only CFP+YFP+RFP+ populations of CEN.PK and Ethanol Red derivatives grown for 35 generations under non-selective conditions are displayed.

## Slide 3
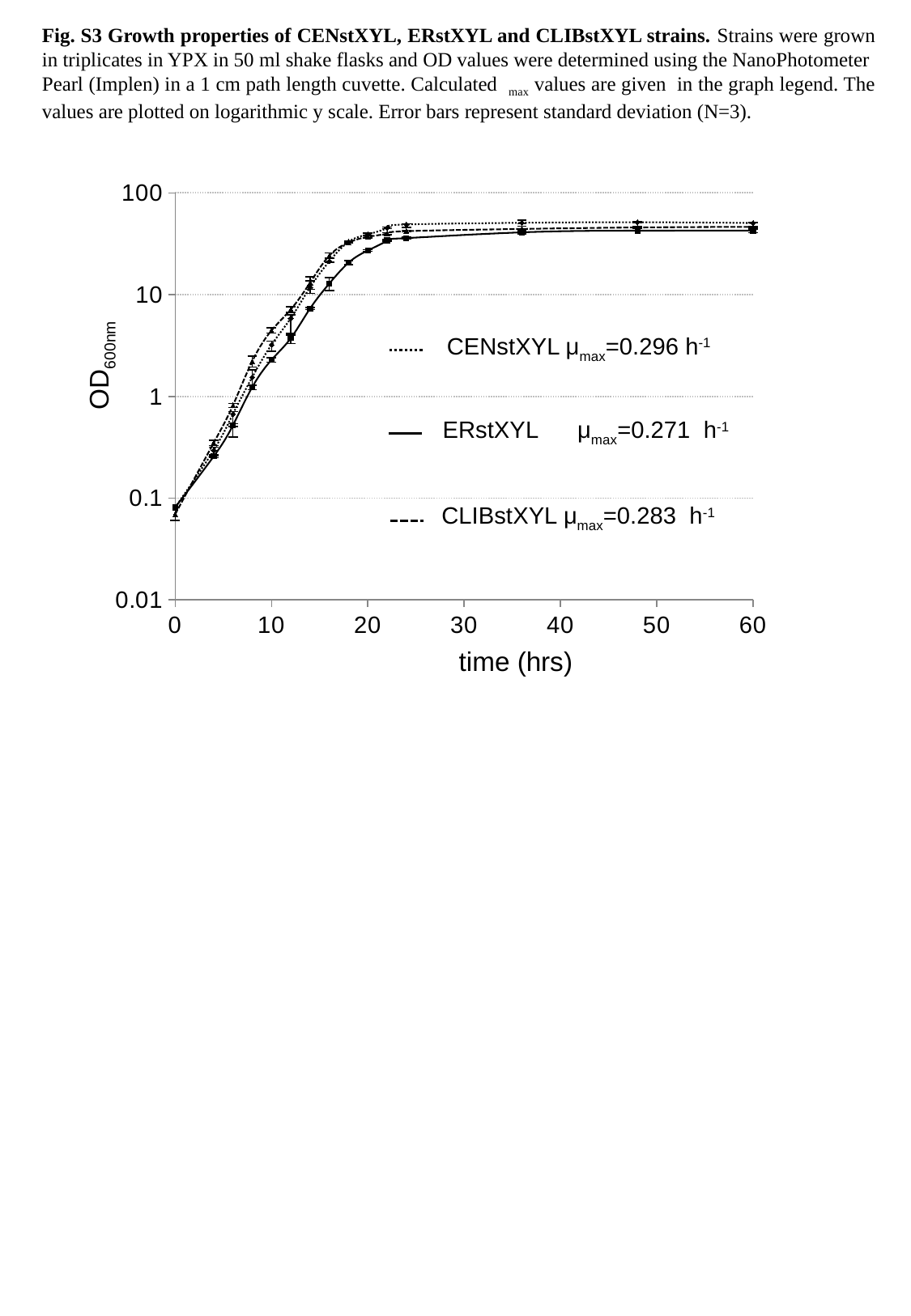

### Chart
| Category | | | |
|---|---|---|---|CENstXYL μmax=0.296 h-1
OD600nm
ERstXYL	 μmax=0.271 h-1
CLIBstXYL μmax=0.283 h-1
time (hrs)

## Slide 4
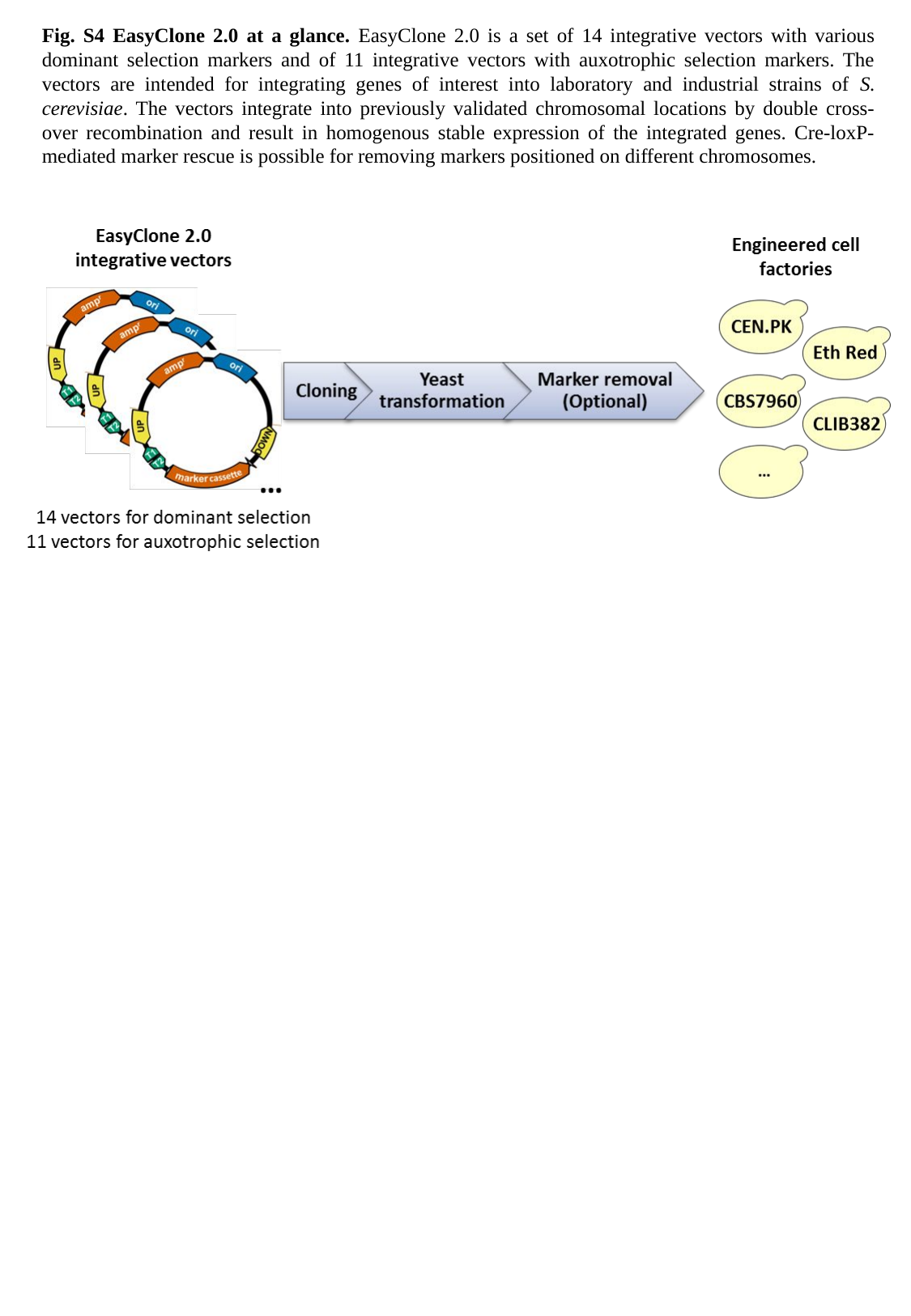

Fig. S4 EasyClone 2.0 at a glance. EasyClone 2.0 is a set of 14 integrative vectors with various dominant selection markers and of 11 integrative vectors with auxotrophic selection markers. The vectors are intended for integrating genes of interest into laboratory and industrial strains of S. cerevisiae. The vectors integrate into previously validated chromosomal locations by double cross-over recombination and result in homogenous stable expression of the integrated genes. Cre-loxP-mediated marker rescue is possible for removing markers positioned on different chromosomes.
