# Supplementary material for: EasyClone 2.0: expanded toolkit of integrative vectors for stable gene expression in industrial Saccharomyces cerevisiae strains
Source: J Ind Microbiol Biotechnol. 2015 Sep 16;42(11):1519–31. doi: 10.1007/s10295-015-1684-8 (PMC4607720; doi:10.1007/s10295-015-1684-8)
Supplement: Supplementary file 2 — Supplementary material 1 (docx 59 kb) [file 10295_2015_1684_MOESM2_ESM.docx]

**Supplementary Material**

EasyClone 2.0: Expanded toolkit of integrative vectors for stable gene expression in industrial *Saccharomyces cerevisiae* strains

Vratislav Stovicek, Gheorghe M. Borja, Jochen Forster, Irina Borodina*

The Novo Nordisk Foundation Center for Biosustainability, Technical University of Denmark, Kogle Allé 6, 2970 Hørsholm, Denmark

*Corresponding author: Irina Borodina, e-mail address: irbo@biosustain.dtu.dk, phone: +45 4525 8020, fax: +45 4525 8001

**Supplementary Tables**

**Table S1 List of vectors used in the study**

| **Basic vectors** | | | |
| --- | --- | --- | --- |
| **Plasmid name** | **Integration site/ replicon** | **Selection marker/insert** | **Source** |
| pX-2 | X-2 | DR-KlURA3 | [1] |
| pX-3 | X-3 | DR-KlURA3 | [1] |
| pX-4 | X-4 | DR-KlURA3 | [1] |
| pXI-1 | XI-1 | DR-KlURA3 | [1] |
| pXI-2 | XI-2 | DR-KlURA3 | [1] |
| pXI-3 | XI-3 | DR-KlURA3 | [1] |
| pXI-5 | XI-5 | DR-KlURA3 | [1] |
| pXII-1 | XII-1 | DR-KlURA3 | [1] |
| pXII-2 | XII-2 | DR-KlURA3 | [1] |
| pXII-4 | XII-4 | DR-KlURA3 | [1] |
| pXII-5 | XII-5 | DR-KlURA3 | [1] |
| pCfB393 | X-2 | LoxP-KlURA3/TEF1p-CFP | [2] |
| pCfB394 | X-3 | LoxP-KlLEU2/TEF1p-RFP | [2] |
| pCfB395 | X-4 | LoxP-SpHIS5/TEF1p-YFP | [2] |
| pCfB2199 | X-3 | LoxP-natMX/TEF1p-GFP | [3] |
| pSH65 | CEN/ARS | ble/GAL1p-Cre | [4] |
| pCfB2047 | Ty2 | LoxP-KlURA3deg | Maury et al., unpublished results |
| pCfB312 | Ty4 | LoxP-KlURA3 | Maury et al., unpublished results |

| **Vectors prepared by site-directed mutagenesis in order to eliminate restriction sites for NheI, PacI, BsaI and BpiI*** | | | | | |
| --- | --- | --- | --- | --- | --- |
| **Plasmid name** | **Integration site** | **Selection marker** | **Template plasmid for mutagenesis** | **Primers for site-directed mutagenesis** | **Source** |
| pCfB2070 | X-4 | DR-KlURA3 | pX-4 | PR7172(128_mutA1437G_fw),  PR7173(128_mutA1437G_rv) | This study |
| pCfB2216 | XI-1 | DR-KlURA3 | pXI-1 | PR7546(p383_mutG4061A_fw),  PR7547(p383_mutG4061A_rv) | This study |
| pCfB2276 | XI-1 | DR-KlURA3 | pCfB2216 | PR7548(p383_mutA4239T_fw),  PR7549 (p383_mutA4239T_rv) | This study |
| pCfB2328 | XI-1 | DR-KlURA3 | pCfB2276 | PR7550(p383_mutC4389T_fw), PR7551(p383_mutC4389T_rv) | This study |
| pCfB2071 | XI-3 | DR-KlURA3 | pXI-3 | PR7174(385_mutA1664G_fw),  PR7175(385_mutA1664G_rv) | This study |
| pCfB2118 | XI-3 | DR-KlURA3 | pCfB2071 | PR7176(385_mutA1794G_fw),  PR7177(385_mutA1794G_rv) | This study |
| pCfB2072 | XII-1 | DR-KlURA3 | pXII-1 | PR7178(129_mutA4338G_fw),  PR7179(129_mutA4338G_rv) | This study |
| pCfB2073 | XII-5 | DR-KlURA3 | pXII-5 | PR7180(131_mutA1825G_fw),  PR7181(131_mutA1825G_rv) | This study |

***** The restriction sites were removed from the vectors in order to make them compatible with MoClo method [5].

| **Vectors prepared by USER cloning** | | | | | | |
| --- | --- | --- | --- | --- | --- | --- |
| **Plasmid name** | **Integrat-ion site** | **Yeast selection marker*** | **Expression cassette** | **Parent vector** | **Cloned BioBricks** | **Source** |
| *EasyClone 2.0 vectors with synthetic auxotrophic selection markers* | | | | | | |
| pCfB2188 | X-2 | LoxP-KlURA3syn | - | - | BB0588, BB0613 | This study |
| pCfB2189 | X-3 | LoxP-KlLEU2syn | - | - | BB0589, BB0614 | This study |
| pCfB2226 | X-4 | LoxP-SpHIS5syn | - | - | BB0590, BB0651 | This study |
| pCfB2374 | XI-1 | LoxP-KlURA3syn | - | - | BB0605, BB0613 | This study |
| pCfB2190 | XI-2 | LoxP-KlLEU2syn | - | - | BB0591, BB0614 | This study |
| pCfB2227 | XI-3 | LoxP-SpHIS5syn | - | - | BB0592, BB0651 | This study |
| pCfB2229 | XI-5 | LoxP-CaLYS5syn | - | - | BB0593, BB0652 | This study |
| pCfB2191 | XII-1 | LoxP-KlURA3syn | - | - | BB0594, BB0613 | This study |
| pCfB2192 | XII-2 | LoxP-KlLEU2syn | - | - | BB0595, BB0614 | This study |
| pCfB2228 | XII-4 | LoxP-SpHIS5syn | - | - | BB0596, BB0651 | This study |
| pCfB2336 | XII-5 | LoxP-SpHIS5syn | - | - | BB0612, BB0651 | This study |
| *EasyClone 2.0 vectors with synthetic dominant selection markers* | | | | | | |
| pCfB2193 | X-2 | LoxP-natMXsyn | - | - | BB0588, BB0598 | This study |
| pCfB2513 | X-2 | LoxP-hphMXsyn | - | - | BB0588, BB0599 | This study |
| pCfB2223 | X-3 | LoxP-kanMXsyn | - | - | BB0589, BB0597 | This study |
| pCfB2194 | X-4 | LoxP-hphMXsyn | - | - | BB0590, BB0599 | This study |
| pCfB2375 | XI-1 | LoxP-natMXsyn | - | - | BB0605, BB0598 | This study |
| pCfB2224 | XI-2 | LoxP-kanMXsyn | - | - | BB0591, BB0597 | This study |
| pCfB2195 | XI-3 | LoxP-hphMXsyn | - | - | BB0592, BB0599 | This study |
| pCfB2196 | XI-5 | LoxP-bleMXsyn | - | - | BB0593, BB0600 | This study |
| pCfB2399 | XI-5 | LoxP-amdSYMsyn | - | - | BB0593, BB0601 | This study |
| pCfB2197 | XII-1 | LoxP-natMXsyn | - | - | BB0594, BB0598 | This study |
| pCfB2400 | XII-1 | LoxP-dsdAsyn | - | - | BB0594, BB1222 | This study |
| pCfB2225 | XII-2 | LoxP-kanMXsyn | - | - | BB0595, BB0597 | This study |
| pCfB2198 | XII-4 | LoxP-hphMXsyn | - | - | BB0596, BB0599 | This study |
| pCfB2337 | XII-5 | LoxP-hphMXsyn | - | - | BB0612, BB0599 | This study |
| *Vectors with expression cassettes encoding fluorescent proteins* | | | | | | |
| pCfB2048 | X-2 | LoxP-kanMXsyn | TEF1p-CFP | - | BB1225, BB0597 | This study |
| pCfB2049 | X-3 | LoxP-natMXsyn | TEF1p-RFP | - | BB1226, BB0598 | This study |
| pCfB2050 | X-4 | LoxP-hphMXsyn | TEF1p-YFP | - | BB1227, BB0599 | This study |
| pCfB2516 | XI-1 | LoxP-natMXsyn | TEF1p-RFP | - | BB1228, BB1230 | This study |
| pCfB2515 | XII-4 | LoxP-hphMXsyn | TEF1p-YFP | - | BB1229, BB1231 | This study |
| pCfB3482 | X-2 | LoxP-natMXsyn | TEF1p-GFP | pCfB2193 | BB1238 | This study |
| pCfB3483 | X-3 | LoxP-kanMXsyn | TEF1p-GFP | pCfB2223 | BB1238 | This study |
| pCfB3484 | X-4 | LoxP-hphMXsyn | TEF1p-GFP | pCfB2194 | BB1238 | This study |
| pCfB3485 | XI-1 | LoxP-natMXsyn | TEF1p-GFP | pCfB2375 | BB1238 | This study |
| pCfB3486 | XI-2 | LoxP-kanMXsyn | TEF1p-GFP | pCfB2224 | BB1238 | This study |
| pCfB3487 | XI-3 | LoxP-hphMXsyn | TEF1p-GFP | pCfB2195 | BB1238 | This study |
| pCfB3488 | XI-5 | LoxP-bleMXsyn | TEF1p-GFP | pCfB2196 | BB1238 | This study |
| pCfB3489 | XII-1 | LoxP-natMXsyn | TEF1p-GFP | pCfB2197 | BB1238 | This study |
| pCfB3490 | XII-2 | LoxP-kanMXsyn | TEF1p-GFP | pCfB2225 | BB1238 | This study |
| pCfB3491 | XII-4 | LoxP-hphMXsyn | TEF1p-GFP | pCfB2198 | BB1238 | This study |
| pCfB3492 | XII-5 | LoxP-hphMXsyn | TEF1p-GFP | pCfB2337 | BB1238 | This study |
| *Vectors for multiple integration into Ty sites* | | | | | | |
| pCfB2404 | Ty2 | LoxP-natMXsyn | - | - | BB1223, BB0598 | This study |
| pCfB2405 | Ty2 | LoxP-hphMXsyn | - | - | BB1223, BB0599 | This study |
| pCfB2401 | Ty4 | LoxP-natMXsyn | - | - | BB1224, BB0598 | This study |
| *Vectors for engineering of xylose utilization* | | | | | | |
| pCfB2871 | Ty2 | LoxP-natMXsyn | PsXYL3←TDH3p-TEF1p→CpXylA | pCfB2404 | BB0464, BB1235, BB1236 | This study |
| pCfB2523 | X-2 | LoxP-hphMXsyn | RPE1←TDH3p-TEF1p→RKI1 | pCfB2513 | BB0464, BB1232, BB1233 | This study |
| pCfB2872 | XI-5 | LoxP-amdSYMsyn | PsTAL1←TDH3p- TEF1p→TKL1 | pCfB2399 | BB0464, BB0263, BB1234 | This study |
| pCfB2874 | XII-2 | LoxP-kanMXsyn | PsSUT1←TDH3p-  TEF1p→CpXylA | pCfB2225 | BB0464, BB1235, BB1237 | This study |
| *Vector carrying bidirectional promoter* | | | | | | |
| p1977 (pUC19-PTDH3-PTEF1) | ori | - | ←TDH3p-TEF1p→ | - | - | This study |

* All the vectors carry amp^R^ cassette for selection in *E. coli*.

**Table S2 List of DNA BioBricks used in this study**. All the BioBricks were generated by PCR.

| **Name** | **Descriptive name** | **Primer pair for PCR** | **Template for PCR** |
| --- | --- | --- | --- |
| BB0588 | X-2 w/o marker | ID401pIntFwdU, ID402pintRevU | pX-2 |
| BB0589 | X-3 w/o marker | ID401pIntFwdU, ID402pintRevU | pX-3 |
| BB0590 | X-4 w/o marker | ID401pIntFwdU, ID402pintRevU | pCfB2070 |
| BB0605 | XI-1_w/o_marker | ID401pIntFwdU, ID402pintRevU | pCfB2328 |
| BB0591 | XI-2 w/o marker | ID401pIntFwdU, ID402pintRevU | pXI-2 |
| BB0592 | XI-3 w/o marker | ID401pIntFwdU, ID402pintRevU | pCfB2118 |
| BB0593 | XI-5 w/o marker | ID401pIntFwdU, ID402pintRevU | pXI-5 |
| BB0594 | XII-1 w/o marker | ID401pIntFwdU, ID402pintRevU | pCfB2072 |
| BB0595 | XII-2 w/o marker | ID401pIntFwdU, ID402pintRevU | pXII-2 |
| BB0596 | XII-4 w/o marker | ID401pIntFwdU, ID402pintRevU | pXII-4 |
| BB0612 | XII-5_w/o_marker | ID401pIntFwdU, ID402pintRevU | pCfB2073 |
| BB0598 | BBnatMX | ID399USERrev, ID400USERfwd | synthetic gene |
| BB0597 | BBkanMX | ID399USERrev, ID400USERfwd | synthetic gene |
| BB0599 | BBhphMX | ID399USERrev, ID400USERfwd | synthetic gene |
| BB0600 | BBble | ID399USERrev, ID400USERfwd | synthetic gene |
| BB0601 | BBamdSYM | ID399USERrev, ID400USERfwd | synthetic gene |
| BB1222 | BBdsdA | ID399USERrev, ID400USERfwd | synthetic gene |
| BB0613 | KlURA3syn_marker | ID399USERrev, ID400USERfwd | synthetic gene |
| BB0614 | KlLEU2syn_marker | ID399USERrev, ID400USERfwd | synthetic gene |
| BB0651 | SpHIS5syn_marker | ID399USERrev, ID400USERfwd | synthetic gene |
| BB0652 | CaLYS5syn_marker | ID399USERrev, ID400USERfwd | synthetic gene |
| BB1223 | p2047 w/o marker | ID401pIntFwdU, ID402pintRevU | pCfB2047 |
| BB1224 | p312 w/o marker | ID401pIntFwdU, ID402pintRevU | pCfB312 |
| BB1225 | p393 w/o marker | ID401pIntFwdU, ID402pintRevU | pCfB393 |
| BB1226 | p394 w/o marker | ID401pIntFwdU, ID402pintRevU | pCfB394 |
| BB1227 | p395 w/o marker | ID401pIntFwdU, ID402pintRevU | pCfB395 |
| BB1228 | TEF1p-RFP | JM235_TADH1_USER_3' , JM237_TCYC1_USER_3' | pCfB394 |
| BB1229 | TEF1p-YFP | JM235_TADH1_USER_3' , JM237_TCYC1_USER_3' | pCfB395 |
| BB1230 | p2375 PCR_open | JM236_TADH1_openvec_USER_3', JM238_TCYC1_openvec_USER_3' | pCfB2375 |
| BB1231 | p2198 PCR_open | JM236_TADH1_openvec_USER_3', JM238_TCYC1_openvec_USER_3' | pCfB2198 |
| BB0464 | <-PTDH3-PTEF1-> | PTEF1->_U2_rv, PTDH3_rv | p1977 (pUC19-PTDH3-PTEF1) |
| BB0263 | TKL1_2 | tkl1_U2_fw, tkl1_U2_rv | *S. cerevisiae* genomic DNA |
| BB1232 | RKI1_2 | RKI1_2FW, RKI1_2REV | *S. cerevisiae* genomic DNA |
| BB1233 | RPE1_1 | RPE1_1FW, RPE1_1REV | *S. cerevisiae* genomic DNA |
| BB1234 | PsTAL1_1 | TAL1_1FW, TAL1_1REV | *P. stipitis* genomic DNA |
| BB1235 | CpXylA_2 | XIclos_2FW, XIclos_2REV | synthetic gene [6] |
| BB1236 | PsXYL3_1 | XYL3_1FW, XYL3_1REV | *P. stipitis* genomic DNA |
| BB1237 | PsSUT1_1 | PsSUT1_1FW, PsSUT1_1RE | *P. stipitis* genomic DNA |
| BB1238 | TEF1p-GFP | pTEF1_fw, GFPopt_rv | pCfB2199 |

**Table S3 List of primers used in this study.** USER compatible ends are underlined. Introduced mutations contained in primer sequence are in underlined bold letters

| **Primer name** | **Sequence** | **Application** |
| --- | --- | --- |
| PR7172(128_mutA1437G_fw) | caagtgactcgag**G**ccacgtggaaag | Site-directed mutagenesis to eliminate BsaI restriction site from vector pX-4 |
| PR7173(128_mutA1437G_rv) | ctttccacgtggc**C**tcgagtcacttg | Site-directed mutagenesis to eliminate BsaI restriction site from vector pX-4 |
| PR7546(p383_mutG4061A_fw) | acaccgccgcgaa**a**actggtcagtggc | Site-directed mutagenesis to eliminate BbsI restriction site from vector pXI-1 |
| PR7547(p383_mutG4061A_rv) | GCCACTGACCAGT**T**TTCGCGGCGGTGT | Site-directed mutagenesis to eliminate BbsI restriction site from vector pXI-1 |
| PR7548(p383_mutA4239T_fw) | agccggaacctgaga**t**gacgccgcgctagaac | Site-directed mutagenesis to eliminate BbsI restriction site from vector pCfB2216 |
| PR7549(p383_mutA4239T_rv) | GTTCTAGCGCGGCGTC**A**TCTCAGGTTCCGGCT | Site-directed mutagenesis to eliminate BbsI restriction site from vector pCfB2216 |
| PR7550(p383_mutC4389T_fw) | gtgagggttgctag**t**tggggcggcggggt | Site-directed mutagenesis to eliminate NheI restriction site from vector pCfB2276 |
| PR7551(p383_mutC4389T_rv) | ACCCCGCCGCCCCA**A**CTAGCAACCCTCAC | Site-directed mutagenesis to eliminate NheI restriction site from vector pCfB2276 |
| PR7174(385_mutA1664G_fw) | ctgtattccttta**g**ttaacgtttttattc | Site-directed mutagenesis to eliminate PacI restriction site from vector pXI-3 |
| PR7175(385_mutA1664G_rv) | gaataaaaacgttaa**c**taaaggaatacag | Site-directed mutagenesis to eliminate PacI restriction site from vector pXI-3 |
| PR7176(385_mutA1794G_fw) | gtcgcatcgct**g**gccagcaaaaag | Site-directed mutagenesis to eliminate NheI restriction site from vector pCfB2071 |
| PR7177(385_mutA1794G_rv) | ctttttgctggc**c**agcgatgcgac | Site-directed mutagenesis to eliminate NheI restriction site from vector pCfB2071 |
| PR7178(129_mutA4338G_fw) | gcaaaagataga**g**gacaaattaatttc | Site-directed mutagenesis to eliminate BbsI restriction site from vector pXII-1 |
| PR7179(129_mutA4338G_rv) | gaaattaatttgtc**c**tctatcttttgc | Site-directed mutagenesis to eliminate BbsI restriction site from vector pXII-1 |
| PR7180(131_mutA1825G_fw) | gtaccggaggag**g**ccgctataac | Site-directed mutagenesis to eliminate BsaI restriction site from vector pXII-5 |
| PR7181(131_mutA1825G_rv) | gttatagcgg**c**ctcctccggtac | Site-directed mutagenesis to eliminate BsaI restriction site from vector pXII-5 |
| ID401pIntFwd | ACCCAAUTCGCCCTATAGTGAGTCG | amplification of EasyClone vector backbone without marker cassette |
| ID402pintRev | ACGCGAUCTTCGAGCGTCCCAAAACC | amplification of EasyClone vector backbone without marker cassette |
| ID399USERrev | ATTGGGUGCATAGGCCACTAGTGGATCTG | amplification of a loxP-flanked marker cassette |
| ID400USERfwd | ATCGCGUCAGCTGAAGCTTCGTACGC | amplification of a loxP-flanked marker cassette |
| XYL3_1FW | AGTGCAGGUAAAACAATGACCACTACCCCATTTGA | amplification of the *P. stipitis* *XYL3* gene for cloning into integrative vector |
| XYL3_1REV | CGTGCGAUTCAGTGTTTCAATTCACTTTCCA | amplification of the *P. stipitis XYL3* gene for cloning into integrative vector |
| TAL1_1FW | AGTGCAGGUAAAACAATGTCCTCCAACTCCCTTGA | amplification of the *P. stipitis TAL1* gene for cloning into integrative vector |
| TAL1_1REV | CGTGCGAUTCAGAATCTGGCTTCCAATTGTT | amplification of the *P. stipitis TAL1* gene for cloning into integrative vector |
| tkl1_U2_fw | ATCTGTCAUAAAACAATGACTCAATTCACTGACATTG | amplification of *S. cerevisiae TKL1* gene for cloning into integrative vector |
| tkl1_U2_rv | CACGCGAUTCAGAAAGCTTTTTTCAAAGGAG | amplification of *S. cerevisiae TKL1* gene for cloning into integrative vector |
| RPE1_1FW | AGTGCAGGUAAAACAATGGTCAAACCAATTATAGC | amplification of *S. cerevisiae RPE1* gene for cloning into integrative vector |
| RPE1_1REV | CGTGCGAUTCAATCTAGCAAATCTCTAGAAC | amplification of *S. cerevisiae RPE1* gene for cloning into integrative vector |
| RKI1_2FW | ATCTGTCAUAAAACAATGGCTGCCGGTGTCCCAAA | amplification of *S. cerevisiae RKI1* gene for cloning into integrative vector |
| RKI1_2REV | CGTGCGAUTCACTTTTCGGTAACTTCAACAC | amplification of *S. cerevisiae RKI1* gene for cloning into integrative vector |
| PsSUT1_1FW | AGTGCAGGUAAAACAATGTCTTCTCAAGATATTCC | amplification of the *P. stipitis SUT1* gene for cloning into integrative vector |
| PsSUT1_1RE | CGTGCGAUTCAAACATGTTCGTCAACAGGCT | amplification of the *P. stipitis SUT1* gene for cloning into integrative vector |
| XIclos_2FW | ATCTGTCAUAAAACAATGAAGAACTACTTCCCAAA | amplification of synthetic *Clostridium phytofermentans* *XylA* gene for cloning into integrative vector |
| XIclos_2REV | CACGCGAUTCATCTGAACAAAATGTTGTTAA | amplification of synthetic Clostridium phytofermentans XylA gene for cloning into integrative vector |
| pTEF1_fw | CGTGCGAUGCACACACCATAGCTTCAAAATG | amplification of TEF1p-GFP biobrick for cloning into integrative vector |
| GFPopt_rv | CACGCGAUTCATTTGTAGAGCTCATCCATGC | amplification of TEF1p-GFP biobrick for cloning into integrative vector |
| PTEF1->_U2_rv | ATGACAGAUTTGTAATTAAAACTTAG | amplification of TDH3p-TEF1p biobrick for cloning into integrative vector |
| PTDH3_rv | ACCTGCACUTTTGTTTGTTTATGTGTGTTTATTC | amplification of TDH3p-TEF1p biobrick for cloning into integrative vector |
| JM236_TADH1_openvec_USER_3' | AGGTCGCUCATCGCACGCA | PCR linearization of integrative vector for USER fusion with a gene expression cassette |
| JM238_TCYC1_openvec_USER_3' | ATCGCGUCAGCTGAAGCTTC | PCR linearization of integrative vector for USER fusion with a gene expression cassette |
| JM235_TADH1_USER_3' | AGCGACCUCATGCTATACCTG | amplification of a gene expression cassette for USER fusion with PCR linearized integrative vector |
| JM237_TCYC1_USER_3' | TGACGCGAUCTTCGAGCG | amplification of a gene expression cassette for USER fusion with PCR linearized integrative vector |
| URA3_DOWN_fw | GGTACCCAATTCGCCCTAGTAAATGCATGTATACTAAACTC | amplification of downstream *URA3* fragment for construction of *URA3* disruption cassette |
| URA3_DOWN_rv | GTTATCAGATATTATCAGGTGG | amplification of downstream *URA3* fragment for construction of *URA3* disruption cassette |
| KanMX_2/3_START_fw | TGGCAATTCCCGGGGATCACGCTGCAGGTCGACAAC | amplification of first 2/3 part of any dominant marker cassette for construction of *URA3* disruption cassette |
| KanMX_2/3_START_rv | AGTGACGACTGAATCCGGTG | amplification of first 2/3 part of kanMX marker cassette for construction of *URA3* disruption cassette |
| KanMX_2/3_END_fw | AATGGGCTCGCGATAATGTC | amplification of last 2/3 part of kanMX marker cassette for construction of *URA3* disruption cassette |
| KanMX_2/3_END_rv | TAGGGCGAATTGGGTACCGCCACTAGTGGATCTGATATCAC | amplification of last 2/3 part of any dominant marker cassette for construction of *URA3* disruption cassette |
| NATfwd | TTCGTGGTCGTCTCGTACT | amplification of last 2/3 part of natMX marker cassette for construction of *URA3* disruption cassette |
| NATrev | GTTGACGTTGGTGACCTC | amplification of first 2/3 part of natMX marker cassette for construction of *URA3* disruption cassette |
| HYGfwd | ACACTACATGGCGTGATTTC | amplification of last 2/3 part of hphMX marker cassette for construction of *URA3* disruption cassette |
| HYGrev | ATTTGTGTACGCCCGACAGT | amplification of first 2/3 part of hphMX marker cassette for construction of *URA3* disruption cassette |
| BLEfwd | CAACCTGCCATCACGAGATT | amplification of last 2/3 part of bleMX marker cassette for construction of *URA3* disruption cassette |
| BLErev | TTCGTTCTGTATCAGGCGCA | amplification of first 2/3 part of bleMX marker cassette for construction of *URA3* disruption cassette |
| amdSYMfwd | GCTCAAGCTAGAGAATTGGA | amplification of last 2/3 part of amdSYM marker cassette for construction of *URA3* disruption cassette |
| amdSYMrev | ACCGTCGAAGTTGTAGTAAC | amplification of first 2/3 part of amdSYM marker cassette for construction of *URA3* disruption cassette |
| DsdAfwd | GATAGCCATTTGCCCATTTC | amplification of last 2/3 part of dsdAMX marker cassette for construction of *URA3* disruption cassette |
| DsDArev | GACAAACAGAGGGTTATCAG | amplification of first 2/3 part of dsdAMX marker cassette for construction of *URA3* disruption cassette |
| ADH1_test_fw | GAAATTCGCTTATTTAGAAGTGTC | verification of a gene expression cassette cloning into EasyClone vectors |
| CYC1_test_rv | CTCCTTCCTTTTCGGTTAGAG | verification of a gene expression cassette cloning into EasyClone vectors |
| ID2220_vec_DW_out | CCTGCAGGACTAGTGCTGAG | verification of EasyClone vector chromosomal integration |
| ID901 X-2-up-out-sq | TGCGACAGAAGAAAGGGAAG | verification of site X-2 chromosomal integration |
| ID902 X-2-down-out-sq | GAGAACGAGAGGACCCAACAT | verification of site X-2 chromosomal integration |
| ID903 X-3-up-out-sq | TGACGAATCGTTAGGCACAG | verification of site X-3 chromosomal integration |
| ID904 X-3-down-out-sq | CCGTGCAATACCAAAATCG | verification of site X-3 chromosomal integration |
| ID905 X-4-up-out-sq | CTCACAAAGGGACGAATCCT | verification of site X-4 chromosomal integration |
| ID906 X-4-down-out-sq | GACGGTACGTTGACCAGAG | verification of site X-4 chromosomal integration |
| ID907 XI-1-up-out-sq | CTTAATGGGTAGTGCTTGACACG | verification of site XI-1 chromosomal integration |
| ID909 XI-2-up-out-sq | GTTTGTAGTTGGCGGTGGAG | verification of site XI-2 chromosomal integration |
| ID910 XI-2-down-out-sq | GAGACAAGATGGGGCAAGAC | verification of site XI-2 chromosomal integration |
| ID911 XI-3-up-out-sq | GTGCTTGATTTGCGTCATTC | verification of site XI-3 chromosomal integration |
| ID912 XI-3-down-out-sq | CACATTGAGCGAATGAAACG | verification of site XI-3 chromosomal integration |
| ID8418 XI-5-up-out-sq | CTCAATGATCAAAATCCTGAATGCA | verification of site XI-5 chromosomal integration |
| ID8419 XI-5-down-out-sq | GCATGGTCACCGCTATCAGC | verification of site XI-5 chromosomal integration |
| ID891 XII-1-up-out-sq | CTGGCAAGAGAACCACCAAT | verification of site XII-1 chromosomal integration |
| ID892 XII-1-down-out-sq | GGACGACAACTACGGAGGAT | verification of site XII-1 chromosomal integration |
| ID893 XII-2-up-out-sq | CGAAGAAGGCCTGCAATTC | verification of site XII-2 chromosomal integration |
| ID894 XII-2-down-out-sq | GGCCCTGATAAGGTTGTTG | verification of site XII-2 chromosomal integration |
| ID895 XII-3-up-out-sq | TGGGCAGCCTTGAGTAAATC | verification of site XII-3 chromosomal integration |
| ID896 XII-3-down-out-sq | TGGCCAATTGTTCAGTCAAG | verification of site XII-3 chromosomal integration |
| ID897 XII-4-up-out-sq | GAACTGACGTCGAAGGCTCT | verification of site XII-4 chromosomal integration |
| ID898 XII-4-down-out-sq | CGTGAAATCTCTTTGCGGTAG | verification of site XII-4 chromosomal integration |
| ID899 XII-5-up-out-sq | CCACCGAAGTTGATTTGCTT | verification of site XII-5 chromosomal integration |
| ID900 XII-5-down-out-sq | GTGGGAGTAAGGGATCCTGT | verification of site XII-5 chromosomal integration |
| ID3108_PTEF1_for_fusion_fw | AGCTACTGAUGCACACACCATAGCTTC | Amplification of *TEF1* promoter |
| PR1565 (PTEF1->_U2_rv) | ATGACAGAUTTGTAATTAAAACTTAG | Amplification of *TEF1* promoter |
| ID3107_PTDH3_for fusion_fw | ATCAGTAGCUATAAAAAACACGCTTTTTCAG | Amplification of *TDH3* promoter |
| PR1853 (PTDH3_rv) | ACCTGCACUTTTGTTTGTTTATGTGTGTTTATTC | Amplification of *TDH3* promoter |
| ALG9_fw | CACGGATAGTGGCTTTGGTGAACAATTAC | qPCR determination of the gene copy number |
| ALG9_rv | TATGATTATCTGGCAGCAGGAAAGAACTTGGG | qPCR determination of the gene copy number |
| CpXylAfw | ACCATTAAGTTGGGTGGTAAGG | qPCR determination of the gene copy number |
| CpXylArv | ATGTAGAAGTCACCGTCGAAAC | qPCR determination of the gene copy number |

**Supplementary Methods**

*Site-directed mutagenesis*

In order to enable application of MoClo technique for the EasyClone 2.0 vectors, we removed several restriction sites (NheI, PacI, BsaI and BbsI) from the vector backbones using site-directed mutagenesis. This was done by performing a PCR reaction on the template vector with mutation-containing primers (Tables S1 & S3). We used Phusion® High-Fidelity DNA Polymerase from New England BioLabs Inc. PCR protocol was as following: initial denaturation at 96°C for 2 min, followed by 18 cycles of [96°C for 50 sec, 55°C for 1 min, 68°C for 6 min], and final extension at 68°C for 20 min. PCR reactions were treated with FastDigest *DpnI* (Life Technologies) at 37°C for 1 hour to digest the methylated template DNA. 5 μl of reaction was transformed into chemically competent *E. coli* cells and the colonies were selected on LB plates supplemented with 100 mg/l ampicillin. The plasmids were isolated and mutations were confirmed by sequencing.

*Construction of vector pCfB1977*

Promoter *P_TEF1_* was PCR amplified from vector pSP-GM1 [7], using primers ID3108_PTEF1_for_fusion_fw and PR1565 (PTEF1->_U2_rv). Promoter *P_TDH3_* was PCR amplified from gDNA of *S. cerevisiae* CEN.PK113-7D using primers ID3107_PTDH3_for fusion_fw and PR1853 (PTDH3_rv). The two fragments were treated with USER^TM^ enzyme (NEB BioLabs), followed by ligation with T4 DNA ligase (Life Technologies). The resulting fused fragment was re-amplified by PCR using primers PR1853 (PTDH3_rv) and PR1565 (PTEF1->_U2_rv), purified from the gel and blunt-ligated into vector pUC19 digested with SmaI. The resulting vector pCfB1977 was confirmed by sequencing.

**Supplementary References**

1. Mikkelsen MD, Buron LD, Salomonsen B, et al. (2012) Microbial production of indolylglucosinolate through engineering of a multi-gene pathway in a versatile yeast expression platform. Metab Eng 14:104–111. doi: 10.1016/j.ymben.2012.01.006

2. Jensen NB, Strucko T, Kildegaard KR, et al. (2014) EasyClone: method for iterative chromosomal integration of multiple genes in *Saccharomyces cerevisiae*. FEMS Yeast Res 14:238–248. doi: 10.1111/1567-1364.12118

3. Stovicek V, Borodina I, Forster J (2015) CRISPR–Cas system enables fast and simple genome editing of industrial *Saccharomyces cerevisiae* strains. Metab Eng Commun 2:13–22. doi: 10.1016/j.meteno.2015.03.001

4. Guldener U, Heck S, Fielder T, et al. (1996) A new efficient gene disruption cassette for repeated use in budding yeast. Nucleic Acids Res 24:2519–2524.

5. Weber E, Engler C, Gruetzner R, et al. (2011) A Modular Cloning System for Standardized Assembly of Multigene Constructs. PLoS ONE 6:e16765. doi: 10.1371/journal.pone.0016765

6. Brat D, Boles E, Wiedemann B (2009) Functional expression of a bacterial xylose isomerase in *Saccharomyces cerevisiae*. Appl Environ Microbiol 75:2304–2311. doi: 10.1128/AEM.02522-08

7. Partow S, Siewers V, Bjørn S, et al. (2010) Characterization of different promoters for designing a new expression vector in *Saccharomyces cerevisiae*. Yeast Chichester Engl 27:955–964. doi: 10.1002/yea.1806
